# Dehydrovomifoliol Alleviates Nonalcoholic Fatty Liver Disease *via* the E2F1/AKT/mTOR Axis: Pharmacophore Modeling and Molecular Docking Study

**DOI:** 10.1155/2023/9107598

**Published:** 2023-02-01

**Authors:** Ji Ma, Ruya Li, Fang Xu, Fang Zhu, Xiangwei Xu

**Affiliations:** ^1^Department of Gastroenterology, The First People's Hospital of Yongkang, Affiliated to Hangzhou Medical College, Jinhua 321300, China; ^2^Department of Pharmacy, The People's Hospital of Jinyun, Lishui 321400, China; ^3^Department of Pharmacy, The First People's Hospital of Yongkang, Affiliated to Hangzhou Medical College, Jinhua 321300, China

## Abstract

**Objective:**

Herbal medicine discovery is a complex and time-consuming process, while pharmacophore modeling and molecular docking methods enable simple and economic studies. The pharmacophore model provides an abstract description of essential intermolecular interactions between chemical structures, and the molecular docking technology can identify novel compounds of therapeutic interests and predict the ligand-target interaction at the molecular level. This study was based on the two methods to elucidate the mechanism of dehydrovomifoliol, an active ingredient extracted from *Artemisia frigida willd*, in nonalcoholic fatty liver disease (NAFLD).

**Methods:**

Bioinformatics analysis was performed to screen target genes of dehydrovomifoliol in NAFLD treatment, which were thus intersected with NAFLD-related differentially expressed genes (DEGs) and NAFLD-related genes. Venn diagram was used to identify candidate DEGs. A pharmacophore model was then generated, and molecular docking was performed. A protein-protein interaction (PPI) network was constructed to identify core genes, which were evaluated using GO and the KEGG enrichment analyses.

**Results:**

Seven target genes of dehydrovomifoliol in NAFLD treatment were screened out, namely E2F1, MERTK, SOX17, MMP9, SULT2A1, VEGFA, and BLVRA. The pharmacophore model and molecular docking of candidate DEGs and dehydrovomifoliol were successfully constructed. E2F1 was identified as a core gene of dehydrovomifoliol in NAFLD treatment. Further enrichment analysis indicated the regulatory role of E2F1 in fat metabolism was associated with the regulation of the AKT/mTOR signaling pathway.

**Conclusion:**

Overall, this study illustrates the anti-NAFLD mechanism of dehydrovomifoliol, which could be a useful compound for developing novel drugs in the treatment of NAFLD.

## 1. Introduction

Nonalcoholic fatty liver disease (NAFLD) is a metabolic disease closely associated with obesity and insulin resistance, leading to dysfunction of adipose tissues and liver [1]. NAFLD distresses one quarter of the worldwide population [2]. Metabolic abnormalities have been the hallmark of the development of NAFLD [3]. Hence, identification of metabolic drivers might reveal therapeutic targets to improve the treatment for NAFLD. The pharmacophore model provides an abstract description of essential intermolecular interactions between chemical structures, which is usually derived both in a receptor-based and in a ligand-based manner [4]. An array of ligand- and structure-based approaches have been developed for improved pharmacophore model building, which have successful and extensive application in de novo design, virtual screening, and lead optimization [5]. Consistently, the molecular docking technology is an *in silico*structure-based method that has been widely used in modern drug design, which can identify novel compounds of therapeutic interests and predict the ligand-target interaction at the molecular level [6]. It is interesting to note a widely applied *in silico* method molecular docking in drug discovery programs, which predicts the binding mode of a given molecule interacting with a specific biological target [7]. Previous studies provided evidence for the feasibility of applying molecular docking approach in studying oxidative stress [8] and key signaling pathways involved in the mechanism of Qushi Huayu Decoction [9] in NAFLD, but these studies fail to single out core genes or illuminate the downstream mechanistic actions. Thus, further investigations are warranted for network pharmacology and molecular docking technology in NAFLD.

Dehydrovomifoliol is an active ingredient extracted from *Artemisia frigida willd*, a traditional Chinese medicine, and can be used as an early lead compound for developing novel drugs for the prevention of NAFLD due to its effects on hepatic lipogenesis and fatty acid oxidation [10]. Multiple kinds of traditional Chinese medicine have demonstrated the ability to regulate E2F transcription factor 1 (E2F1); for instance, Jieduan-Niwan formula can protect the liver of acute-on-chronic liver failure rats by inhibiting E2F transcription factor 1 (E2F1)-mediated apoptotic signaling pathway [11]. However, the possible regulation of dehydrovomifoliol on E2F1 has not been characterized and thus becomes the focus of this study. E2F1, a transcription factor, contributes to the development of liver pathology due to its regulatory role in lipid synthesis and glycolysis while its deletion has been shown to abrogate hepatic steatosis in a murine model of NAFLD [12]. Of interest, E2F1 has been reported to function as a critical antiapoptotic factor in human and rodent liver cancer *via* activation of the protein kinase B (AKT)/mammalian target of rapamycin (mTOR) signaling pathway [13]. In addition, inhibiting the AKT/mTOR signaling pathway reduces lipid accumulation and lipogenesis, while increasing fatty acids oxidation and very low density lipoprotein assembly and secretion in goose hepatocytes [14]. Therefore, the effect of dehydrovomifoliol may be associated with the E2F1/AKT/mTOR axis. In lieu of the abovementioned findings and evidence, the present study mainly centered on elaborating the mechanisms underlying the role of dehydrovomifoliol in NAFLD by combining the pharmacophore modeling and molecular docking approach.

## 2. Materials and Methods

### 2.1. Retrieval of Target Genes of Dehydrovomifoliol

The 3D chemical structure map of dehydrovomifoliol was downloaded from the PubChem database and saved in “SDF” format. The SDF file of dehydrovomifoliol was uploaded to the PharmMapper server, after which dehydrovomifoliol was simulated for molecular-target protein docking using the inverse docking tool PharmMapper server, with “Human Protein Targets Only” set as the target range and the remaining parameters were kept at their default values.

### 2.2. Acquisition of High-Throughput Transcriptome Sequencing Dataset

NAFLD-related gene expression datasets GSE73500 and GSE89632 were downloaded from the GEO database. The GSE73500 dataset includes three liver tissue samples from NAFLD rats and three control liver tissue samples. The GSE89632 dataset includes nineteen liver tissue samples from NAFLD rats and 24 control liver tissue samples.

### 2.3. Quality Control and Reference Genome Alignment of Datasets

The quality of paired-end reads of raw sequencing data was assessed using the FastQC v0.11.8 software. Preprocessing of raw data was performed by using the Cutadapt v1.18 software: the illumina sequencing adaptors and poly (A) tail sequences were removed. The reads with an N content of over 5% were removed using a Perl script. The reads with a 70% base mass above 20 were extracted using FASTX Toolkit software (v0.0.13). Next, two-end sequences were repaired using BBMap software. Finally, the filtered high-quality reads fragment human reference genome was aligned using HISAT2 software (v0.7.12).

### 2.4. Differential Expression Analysis

With normal samples as the control, the R “limma” package (version 3.4.1) was applied for differential analysis to identify the differentially expressed mRNAs with |log2FC| > 1 and *p* value < 0.05 as the threshold.

### 2.5. NAFLD-Related Database Retrieval

NAFLD-related target genes were retrieved from the GeneCards database with “nonalcoholic fatty liver disease” and “fat cell proliferation” as the key words. With Relevance Score  ≥ 1 as the screening criteria, more relevant targets were obtained for subsequent analysis.

### 2.6. Venn Analysis

The Draw Venn Diagram tool was used to perform Venn analysis on the dataset analysis results and the retrieval results of the GeneCards database to identify candidate genes.

### 2.7. Construction of Candidate Gene-Protein Interaction Networks

The Cytoscape (v3.8.2) plug-in Bisogenet and cytoNCA were used for intersection analysis. Bisogenet provides database information, while cytoNCA provides attribute information of nodes after network composition, and is utilized to calculate the degree centrality (DC) of nodes and observe the core genes intuitively. A protein-protein interaction (PPI) network was constructed and topological analysis was performed. Core targets were screened according to the DC and the betweenness centrality (BC).

Through the STRING database, the interaction network of E2F1-mediated genes related to fat metabolism, was obtained with the “*Homo sapiens*” as the species, which was then imported into Cytoscape software (v3.8.2) for analysis and ranking. The Degree value and Combine score value were represented by color, and the candidate genes were ranked according to the Degree value.

### 2.8. Molecular Docking Simulation

The crystal structures of seven core proteins E2F1 (PDB ID : 1O9K), MERTK (PDB ID : 2P0C), SOX17 (PDB ID : 2YUI), MMP9 (PDB ID : 1ITV), SULT2A1 (PDB ID : 2QP4), VEGFA (PDB ID : 1BJ1), and BLVRA (PDB ID : 2H63) were downloaded and screened from the Protein Data Bank. The structure of dehydrovomifoliol was downloaded from the PubChem database, converted to a 3D structure using Chem3DUltra 14.0 software, and subjected to energy minimization using the MM2 algorithm. The receptors of the seven target proteins were then subjected to dehydration and organic substance removal using PyMOL software. Target protein receptor molecules were hydrogenated, followed by charge calculation using AutoDockTools 1.5.6 software. Next, compounds and target protein receptors were converted into “PDBQT” files with appropriate box center and box lattice parameters set. Finally, Vina 1.5.6 software was run to evaluate the molecular docking and calculate the docking energy value.

### 2.9. Functional Enrichment Analysis of Candidate Genes

The R “ClusterProfiler” package was used for GO enrichment analysis, which consists of biological process (BP), molecular function (MF), and cellular component (CC), with *p* < 0.05 as the threshold. KEGG enrichment analysis was performed to analyze cellular functions and signaling pathways mainly affected by the potential targets and key targets with *p* < 0.05 as the threshold.

### 2.10. Statistical Analysis

All statistical analyses in this study were performed using SPSS 21.0 software (IBM Corp. Armonk, NY). Measurement data were presented as mean ± standard deviation. Data obeying normal distribution and homogeneity of variance between two groups were compared using unpaired *t* test. A *p* value < 0.05 was considered significant.

## 3. Results

### 3.1. Bioinformatics Analysis-Based Identification of Seven Target Genes of Dehydrovomifoliol in NAFLD Treatment

Dehydrovomifoliol is an active ingredient extracted from the traditional Chinese medicine *Artemisia frigida willd*. This study explored the mechanism of dehydrovomifoliol in NAFLD based on pharmacophore model building and molecular docking methods. The experimental schedule is shown in Figure S1.

**Figure 1:**
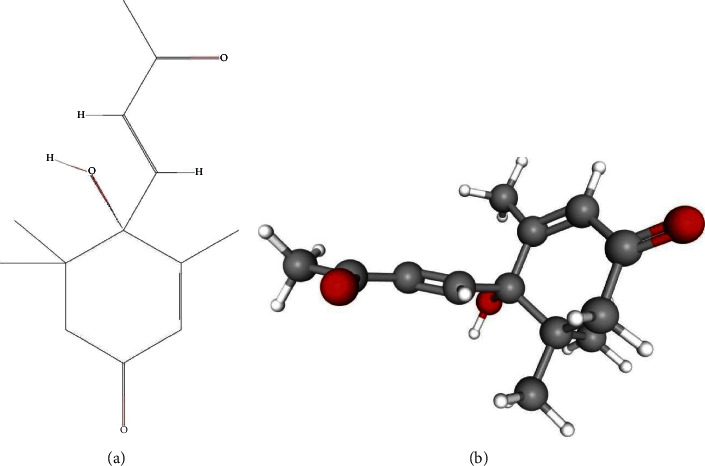
Chemical structure of dehydrovomifoliol analyzed by using the PubChem database. (a) 2D chemical structure of dehydrovomifoliol. (b) 3D chemical structure of dehydrovomifoliol.

**Figure 2:**
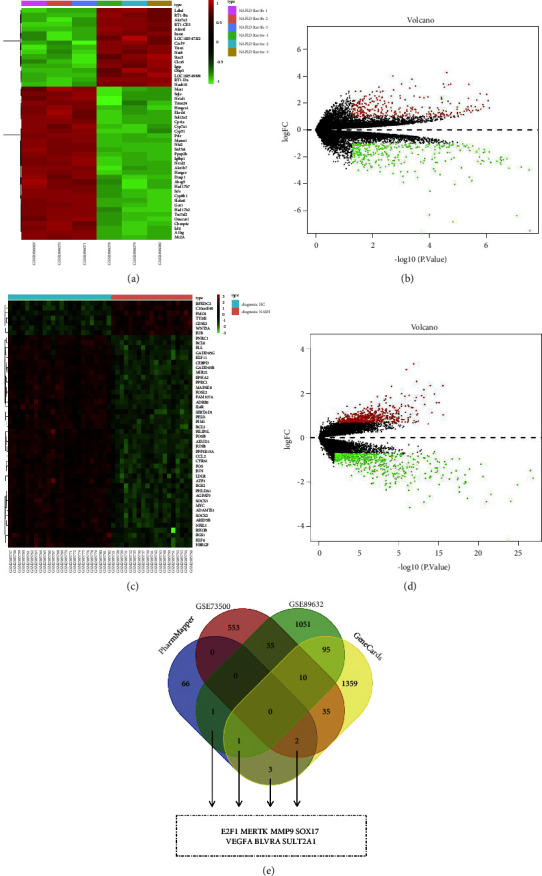
Bioinformatics analysis-based prediction of target genes of dehydrovomifoliol involved in NAFLD treatment. (a) A heat map of the expression of DEGs in the GSE73500 dataset. (b) A volcano plot of the expression of DEGs in the GSE73500 dataset. Green dots indicate downregulated genes, red dots indicate upregulated genes, and gray dots indicate genes with no significant difference. (c) A heat map of the expression of DEGs in the GSE89632 dataset. (d) A volcano plot of the expression of DEGs in the GSE89632 dataset. Green dots indicate downregulated genes, red dots indicate upregulated genes, and gray dots indicate genes with no significant difference. (e) Venn diagram of the target genes from the PharmMapper database, DEGs from the GSE73500 and GSE89632 datasets, and NAFLD-related genes from the GeneCards database. Seven target genes of dehydrovomifoliol in NAFLD treatment were obtained.

First, the chemical structure of dehydrovomifoliol was determined using the PubChem database (Figures 1(a) and 1(b)). The PharmMapper server was then used to analyze and screen the target genes of dehydrovomifoliol, with 73 related genes obtained. Differential analysis of the GSE73500 dataset yielded 635 differentially expressed genes (DEGs), including 305 upregulated DEGs and 330 downregulated DEGs (Figures 2(a) and 2(b)). In addition, 1193 DEGs were obtained from the GSE89632 dataset, including 579 upregulated DEGs and 614 downregulated DEGs (Figures 2(c) and 2(d)). From the GeneCards database, 1505 NAFLD-related genes were identified with Relevance Score ≥ 1 as the screening criteria.

Subsequently, the target genes from the PharmMapper database, DEGs from the GSE73500 and GSE89632 datasets, and NAFLD-related genes from the GeneCards database were subjected to Venn analysis, with seven target genes of dehydrovomifoliol in NAFLD treatment obtained (Figure 2(e)), namely E2F1, MERTK, SOX17, MMP9, SULT2A1, VEGFA, and BLVRA.

### 3.2. Construction of Pharmacophore Models for Candidate DEGs

**Figure 3:**
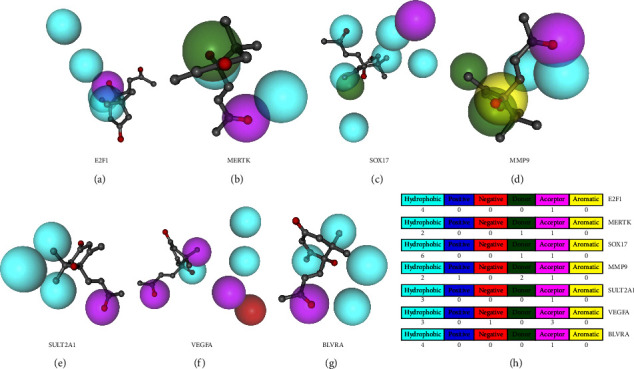
Pharmacophore structure analysis of dehydrovomifoliol and the seven receptor targets involved in NAFLD treatment using the PharmMapper database. (a) Pharmacophore models for the interaction of E2F1 with dehydrovomifoliol. (b) Pharmacophore models for the interaction of MERTK with dehydrovomifoliol. (c) Pharmacophore models for the interaction of SOX17 with dehydrovomifoliol. (d) Pharmacophore models for the interaction of MMP9 with dehydrovomifoliol. (e) Pharmacophore models for the interaction of SULT2A1 with dehydrovomifoliol. (f) Pharmacophore models for the interaction of VEGFA with dehydrovomifoliol. (g) Pharmacophore models for the interaction of BLVRA with dehydrovomifoliol. (h) Pharmacophore models for the interaction of E2F1, MERTK, SOX17, MMP9, SULT2A1, VEGFA, and BLVRA with dehydrovomifoliol.

When drug molecules interact with receptor targets, it is necessary to generate geometric and energy matched active conformations with targets. Pharmacophore can represent the characteristic elements of pharmacodynamics and their spatial arrangement in the active molecule that play an important role in the activity and can also reflect the active site where the ligand and the receptor interact. Pharmacophore structure analysis of dehydrovomifoliol and the seven receptor targets involved in NAFLD treatment was performed using the PharmMapper database. The results showed that four hydrophobic and one acceptor interact with E2F1; two hydrophobic, one donor, and one acceptor interact with MERTK; six hydrophobic, one donor, and one acceptor interact with SOX17; two hydrophobic, one positive region, two donor, and one acceptor interact with MMP9; three hydrophobic and one acceptor interact with SULT2A1; three hydrophobic, one negative region, and three acceptor interact with VEGFA; four hydrophobic and one acceptor interact with BLVRA (Figures 3(a)–3(h)). Accordingly, pharmacophore models for the interaction of candidate genes with dehydrovomifoliol were constructed for subsequent analysis.

### 3.3. Validation of the Molecular Docking between Candidate Proteins and Dehydrovomifoliol

**Figure 4:**
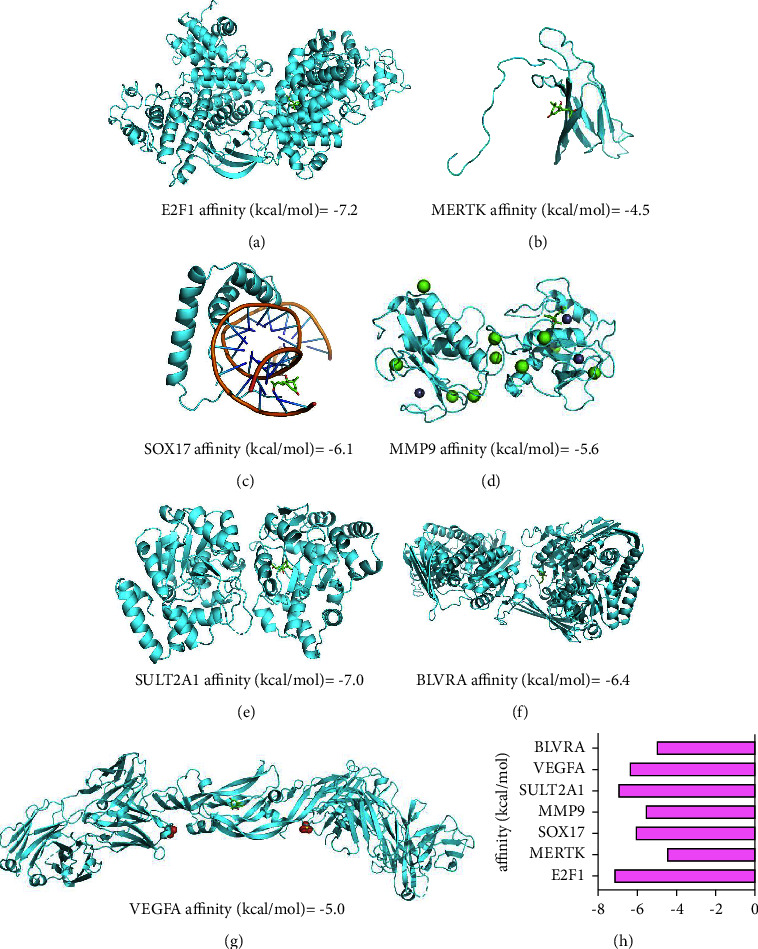
Molecular docking analysis of candidate proteins and dehydrovomifoliol using AutoDockTools 1.5.6 and Vina 1.5.6 software. (a) Molecular docking of E2F1 and dehydrovomifoliol. (b) Molecular docking of MERTK and dehydrovomifoliol. (c) Molecular docking of SOX17 and dehydrovomifoliol. (d) Molecular docking of MMP9 and dehydrovomifoliol. (e) Molecular docking of SULT2A1 and dehydrovomifoliol. (f) Molecular docking of VEGFA and dehydrovomifoliol. (g) Molecular docking of BLVRA and dehydrovomifoliol. (h) A bar graph of the affinity of E2F1, MERTK, SOX17, MMP9, SULT2A1, VEGFA, and BLVRA to dehydrovomifoliol. It was noted that E2F1 had the highest binding free energy to dehydrovomifoliol compared to the other protein molecules.

Molecular docking analysis of candidate proteins and dehydrovomifoliol was performed using AutoDockTools 1.5.6 and Vina 1.5.6 software. When the binding energy is < 0 kJ/mol, the molecular proteins are considered to bind spontaneously and interact with each other; the lower binding energy reflects more stable molecular conformation. The results of docking analysis of candidate proteins with dehydrovomifoliol are shown in Figures 4(a)–4(h). The docking results of the seven candidate proteins and dehydrovomifoliol are displayed in 3D plots, which can visually demonstrate the binding mode of target protein receptors and compounds, as well as the interaction with the surrounding amino acid residues. The results revealed that E2F1 had the highest binding free energy to dehydrovomifoliol compared to the other protein molecules, indicating that dehydrovomifoliol may participate in related mechanistic responses by regulating E2F1 protein in NAFLD treatment.

### 3.4. E2F1 Is a Core Gene of Dehydrovomifoliol in NAFLD Treatment

**Figure 5:**
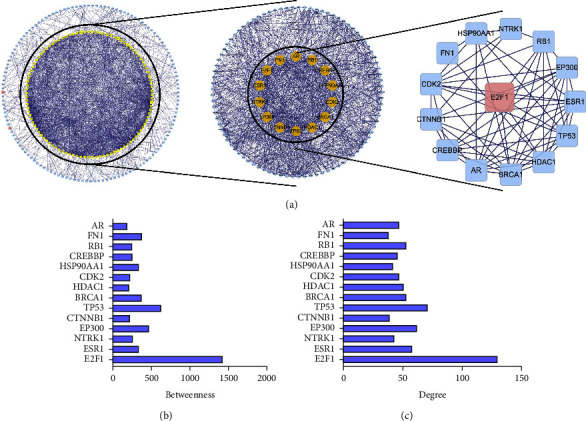
Screening of the core gene of dehydrovomifoliol in NAFLD treatment. PPI network analysis was performed using the Cytoscape plug-in Bisogenet to analyze the network of seven candidate genes. (a) Screening of core genes of dehydrovomifoliol in NAFLD treatment by using the Cytoscape plug-in Bisogenet and CytoNCA. (b) BC of the screened core genes of dehydrovomifoliol in NAFLD treatment. (c) Degree of the screened core genes of dehydrovomifoliol in NAFLD treatment. It was noted that E2F1 ranked the first in both BC and Degree, and it was thus selected as the core gene.

Seven candidate genes were imported into Cytoscape software to build a network map, whereupon PPI network analysis was performed using the Cytoscape plug-in Bisogenet. HPRD, BIND, DIP, MINT, INTACT, and BIOGRID databases were selected for analysis, and 238 nodes and 1509 edges were obtained. The DC values of the 238 target proteins were calculated using the plug-in CytoNCA, and the data with DC values > 10 were selected to create a subnetwork, with 103 nodes and 1047 edges determined. The plug-in CytoNCA was then used for topological analysis to calculate the BC of 103 target proteins in the subnetwork, and a total of 14 target proteins of BC ≥ 200 were screened. A PPI network was created, resulting in 14 nodes and 59 edges (Figure 5(a)). Among them, E2F1 ranked the first in both BC and Degree (Figures 5(b) and 5(c)), and E2F1 was thus selected as the core gene of dehydrovomifoliol in NAFLD treatment for subsequent experiments.

### 3.5. E2F1 Is Involved in the Fat Metabolism during NAFLD

**Figure 6:**
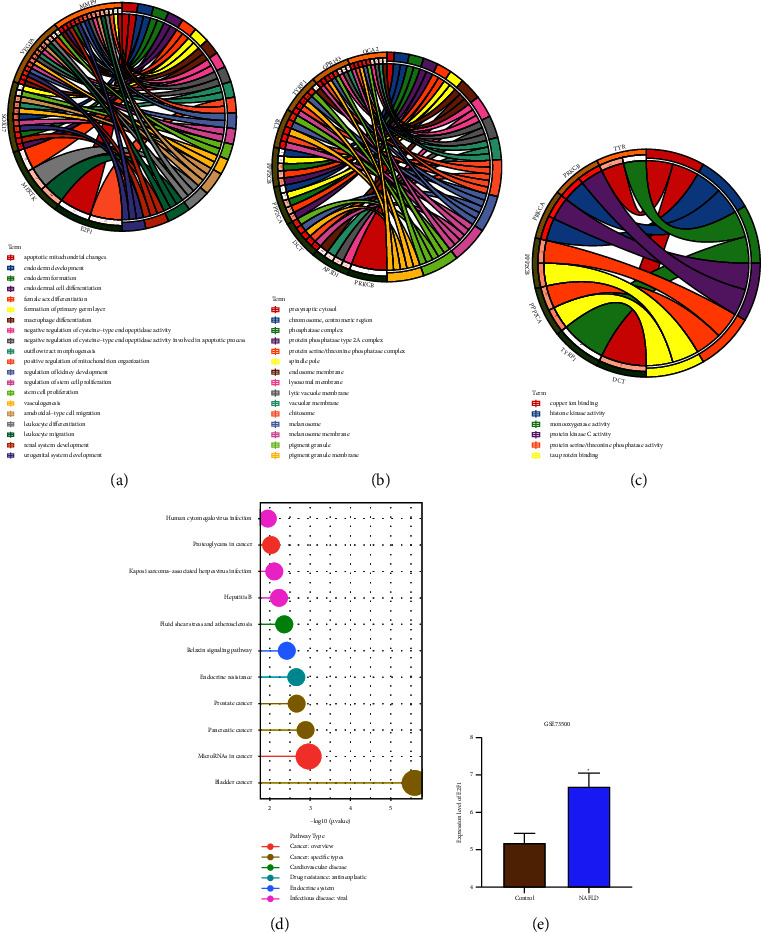
GO and KEGG enrichment pathway analyses of the candidate genes. (a) BP of GO functional enrichment analysis of candidate DEGs. (b) CC of GO functional enrichment analysis of candidate DEGs. (c) MF of GO functional enrichment analysis of candidate DEGs. (d) KEGG pathway enrichment analysis of candidate DEGs. Dot size indicates the number of the selected genes, and color represents the *p* value of enrichment analysis. (e) Expression of E2F1 in the liver tissue samples from NAFLD rats and control liver tissue samples in the GSE73500 dataset. E2F1 was significantly highly expressed in rats with NAFLD. ^*∗*^*p* < 0.05.

GO enrichment analysis revealed that the seven candidate DEGs were mainly enriched in the BP of fat cell proliferation, regulation of fat cell proliferation, and negative regulation of cysteine-type endopeptidase activity involved in apoptotic process entries (Figure 6(a)); CC of protein phosphatase type 2A complex, protein serine/threonine phosphatase complex, and phosphatase complex (Figure 6(b)); and MF of histone kinase activity, protein kinase C activity, tau protein binding and protein serine/threonine phosphatase activity (Figure 6(c)). Additionally, the results of KEGG pathway enrichment analysis showed that the seven candidate DEGs were chiefly involved in the endocrine resistance, relaxin signaling pathway, fluid shear stress and atherosclerosis, and hepatitis B (Figure 6(d)).

During the development of NAFLD, insulin resistance leads to increased liver lipogenesis and inhibits fat decomposition, resulting in fat storage in adipocytes in the form of triglycerides in the liver and the consequent formation of fatty liver [15]. Among the seven candidate DEGs, E2F1 can participate in the regulation of fat cell proliferation and fat storage, and plays an important regulatory role in the development of NAFLD. Moreover, differential analysis of the GSE73500 dataset suggested that E2F1 was significantly highly expressed in rats with NAFLD (Figure 6(e)).

### 3.6. E2F1 Regulates Fat Metabolism by Mediating the AKT/mTOR Signaling Pathway

**Figure 7:**
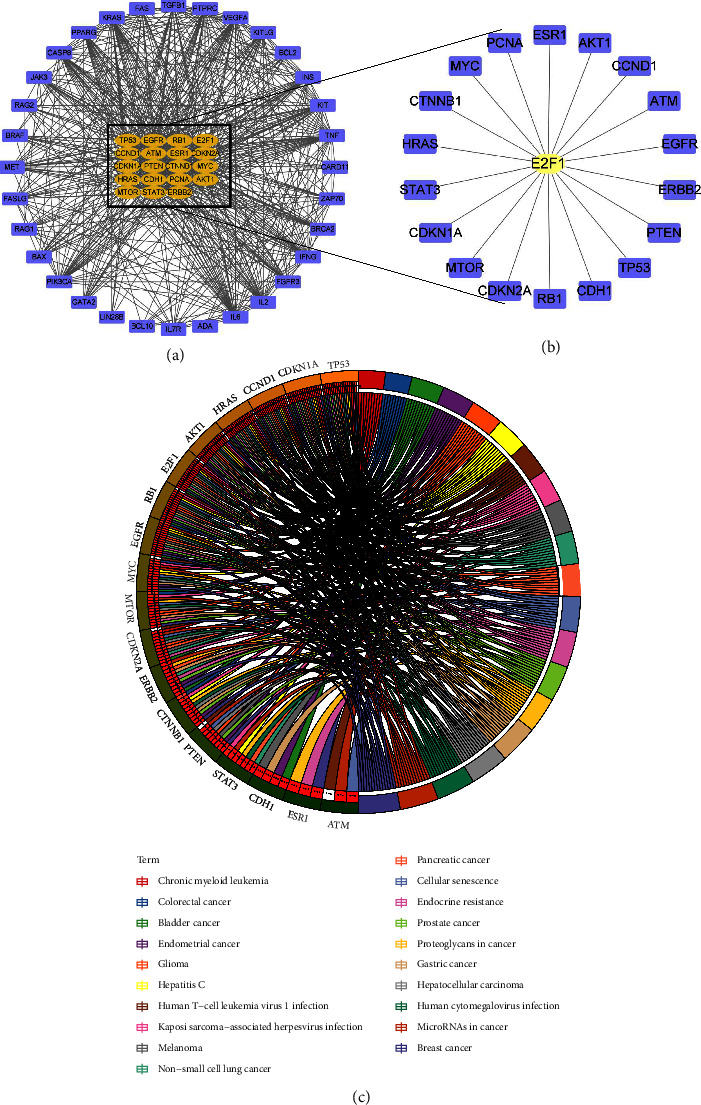
Screening of genes regulated by E2F1 during NAFLD development. (a) A PPI network of fat cell proliferation-related genes and E2F1. (b) Screening of fat cell proliferation genes related to the core gene E2F1. (c) KEGG enrichment analysis of fat metabolism genes related to the core gene E2F1.

The accumulation of fat in liver tissues leads to an increase of free fatty acids and reactive oxygen species, which can cause oxidative stress and endoplasmic reticulum stress, further resulting in damage to hepatocytes and aggravating NAFLD [16]. To explore the regulation of fat metabolism by E2F1, related genes were retrieved from the GeneCards website with “fat cell proliferation” as the key word. The top 50 fat cell proliferation-related genes and E2F1 were selected to construct a PPI network, and 693 edges were obtained, with the PPI enrichment *p* value < 1.0*e* − 16 (Figure 7(a)). Then, 19 fat metabolism genes related to the core gene E2F1 were screened (Figure 7(b)). Further KEGG enrichment analysis suggested that the 19 genes were involved in the regulation of FoxO signaling pathway, EGFR tyrosine kinase inhibitor resistance, and ErbB signaling pathway (Figure 7(c)). Among them, the AKT/mTOR signaling pathway participates in various mechanisms related to fat metabolism, such as regulation of fatty acid *β*-oxidation, fatty acid catabolic processes, fat cell differentiation, and fatty acid oxidation (Table S1). Evidence has shown that activation of the AKT/mTOR signaling pathway is closely related to oxidative stress and fat metabolism in NAFLD [17, 18]. Therefore, we speculated that E2F1 can regulate fat metabolism and participate in NAFLD development by mediating the AKT/mTOR signaling pathway.

## 4. Discussion

Traditional Chinese medicine have gained widespread attention in recent years, but knowledge of the mechanisms by which they act is still very limited. In this work, we generated a qualitative pharmacophore model and applied the molecular docking technology to elucidate the mechanism underlying dehydrovomifoliol alleviating NAFLD. The results demonstrated that dehydrovomifoliol may alter abnormalities in fat metabolism and consequently alleviate NAFLD by downregulating the E2F1/AKT/mTOR axis.

The results of the current study identified seven target genes of dehydrovomifoliol in NAFLD treatment, namely E2F1, MERTK, SOX17, MMP9, SULT2A1, VEGFA, and BLVRA. Among them, MERTK [19], MMP9 [20], and VEGFA [21] have been reported to be involved in NAFLD, although the mechanistic basis remains unclear. Besides, SOX17 [22], SULT2A1 [23], and BLVRA [24] have potential roles to play in other liver diseases. Limited evidence exists regarding their involvement in the pharmacological mechanism of traditional Chinese medicine in NAFLD. However, a prior study has documented that traditional Chinese medicine may confer effects by regulating E2F1. For instance, Jieduan-Niwan formula alleviates acute-on-chronic liver failure by repressing inhibiting E2F1-mediated apoptotic signaling pathways [11].

It is interesting to note that molecular docking, as a widely applied *in silico* method in drug discovery programs, can predict the binding mode of a given molecule interacting with a specific biological target [7]. Subsequent pharmacophore model building and molecular docking method in this study revealed that E2F1 was a core gene of dehydrovomifoliol in NAFLD treatment. E2F1 is known as a key regulator of cell cycle, DNA-damage response, and apoptosis, and it plays a crucial role in the control of metabolism [25]. Additionally, E2F1 deletion has been shown to abrogate hepatic steatosis in different mouse models of NAFLD [12]. The current results also indicated the involvement of E2F1 in the fat metabolism during NAFLD. In line with this, a recent study has identified the increased expression of E2F1 in human NAFLD, and that increased E2F1 can function as a metabolic driver of NAFLD-related hepatocellular carcinoma [26]. Nonetheless, further in-depth investigation is required owing to the insufficient evidence to support the correlation of dehydrovomifoliol with E2F1.

Further mechanistic investigations suggested that E2F1 may regulate fat metabolism and then participate in the development of NAFLD by mediating the AKT/mTOR signaling pathway. Additionally, the E2F1/DDX11 axis is capable of promoting malignant behaviors of hepatocellular carcinoma cells *via* activation of the PI3K/AKT/mTOR signaling pathway [27]. Accumulating evidence suggests a close association of AKT/mTOR signaling pathway activation with oxidative stress and metabolism in NAFLD [17, 18]. Recent data highlighted that salvianolic acid A, a traditional Chinese medicine extracted from Danshen, may curtail the process of liver fibrosis by regulating the PI3K/AKT/mTOR signaling pathway [28]. Thus, studying the E2F1/AKT/mTOR axis maybe beneficial to the mechanistic understanding of traditional Chinese medicine and the development of new therapeutic strategies for NAFLD.

## 5. Conclusion

**Figure 8:**
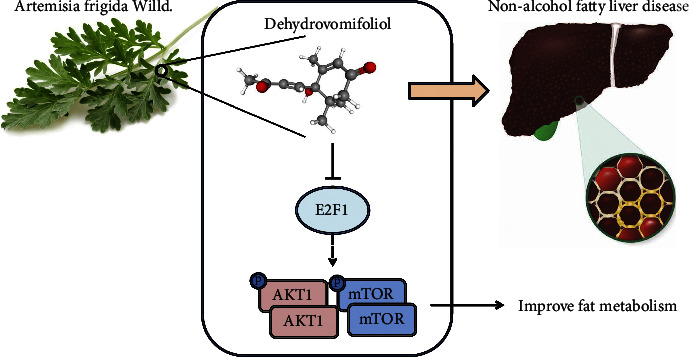
Schematic representation summarizing the role of dehydrovomifoliol in NAFLD. Dehydrovomifoliol downregulates E2F1 to inactivate the AKT/mTOR signaling pathway, thereby improving fat metabolic abnormalities and alleviating NAFLD.

In summary, the key findings from the present study suggested that dehydrovomifoliol potentially alleviates NAFLD through disruption of the E2F1/AKT/mTOR axis (Figure 8). These results are very helpful to provide clues and useful exploration for drug research based on natural products, especially dehydrovomifoliol. Further studies based on clinical cohorts and *in vitro* and *in vivo* experiments are required to substantiate the mechanistic basis and therapeutic effects of dehydrovomifoliol in NAFLD.

## Data Availability

The datasets used and/or analyzed during the current study are available from the corresponding author on reasonable request.

## Abbreviations

NAFLD: Nonalcoholic fatty liver disease
E2F1: E2F transcription factor 1
AKT: Activation of the protein kinase B
mTOR: Mammalian target of rapamycin
DC: Degree centrality
PPI: Protein-protein interaction
BC: Betweenness centrality
BP: Biological process
MF: Molecular function
CC: Cellular component
DEG: Differentially expressed gene
MERTK: MER proto-oncogene, tyrosine kinase
SOX17: SRY (sex determining region Y)-box 17
MMP9: Matrix metallopeptidase 9
SULT2A1: Sulfotransferase family 2A member 1
VEGFA: Vascular endothelial growth factor A
BLVRA: Biliverdin reductase A
GEO: Gene Expression Omnibus
KEGG: Kyoto Encyclopedia of Genes and Genomes.

## Supplementary Materials

Supplementary Materials Figure S1: protocols of the present study. Table S1: KEGG enrichment analysis of fat metabolism genes related to the core gene E2F1.
