# Supplementary material for: Dehydrovomifoliol Alleviates Nonalcoholic Fatty Liver Disease via the E2F1/AKT/mTOR Axis: Pharmacophore Modeling and Molecular Docking Study
Source: Evid Based Complement Alternat Med. 2023 Feb 1;2023:9107598. doi: 10.1155/2023/9107598 (PMC9908351; doi:10.1155/2023/9107598)
Supplement: Supplementary Materials — Figure S1: protocols of the present study. Table S1: KEGG enrichment analysis of fat metabolism genes related to the core gene E2F1. [file 9107598.f1.docx]

**Table S1** KEGG enrichment analysis of fat metabolism genes related to the core gene E2F1

| ID | Description | *p* value | gene ID | |
| --- | --- | --- | --- | --- |
| GO:0045444 | fat cell differentiation | 7.69E-05 | MTOR/AKT1/CCND1/E2F1 | |
| GO:0031998 | regulation of fatty acid β-oxidation | 0.000161 | MTOR/AKT1 | |
| GO:0045598 | regulation of fat cell differentiation | 0.00034 | MTOR/AKT1/E2F1 | |
| GO:0046320 | regulation of fatty acid oxidation | 0.000456 | | MTOR/AKT1 |
| GO:0071398 | cellular response to fatty acid | 0.001586 | AKT1/E2F1 | |
| GO:0006635 | fatty acid β-oxidation | 0.002196 | MTOR/AKT1 | |
| GO:0070542 | response to fatty acid | 0.003611 | AKT1/E2F1 | |
| GO:0019395 | fatty acid oxidation | 0.004305 | MTOR/AKT1 | |
| GO:0009062 | fatty acid catabolic process | 0.004396 | MTOR/AKT1 | |
| GO:0019217 | regulation of fatty acid metabolic process | 0.004488 | MTOR/AKT1 | |
| GO:0046322 | negative regulation of fatty acid oxidation | 0.010559 | AKT1 | |
| GO:0070341 | fat cell proliferation | 0.010559 | E2F1 | |
| GO:0070344 | regulation of fat cell proliferation | 0.010559 | E2F1 | |
| GO:0021781 | glial cell fate commitment | 0.014753 | CTNNB1 | |
| GO:1902001 | fatty acid transmembrane transport | 0.015799 | AKT1 | |
| GO:0090335 | regulation of brown fat cell differentiation | 0.016844 | MTOR | |
| GO:2000191 | regulation of fatty acid transport | 0.028267234 | AKT1 | |


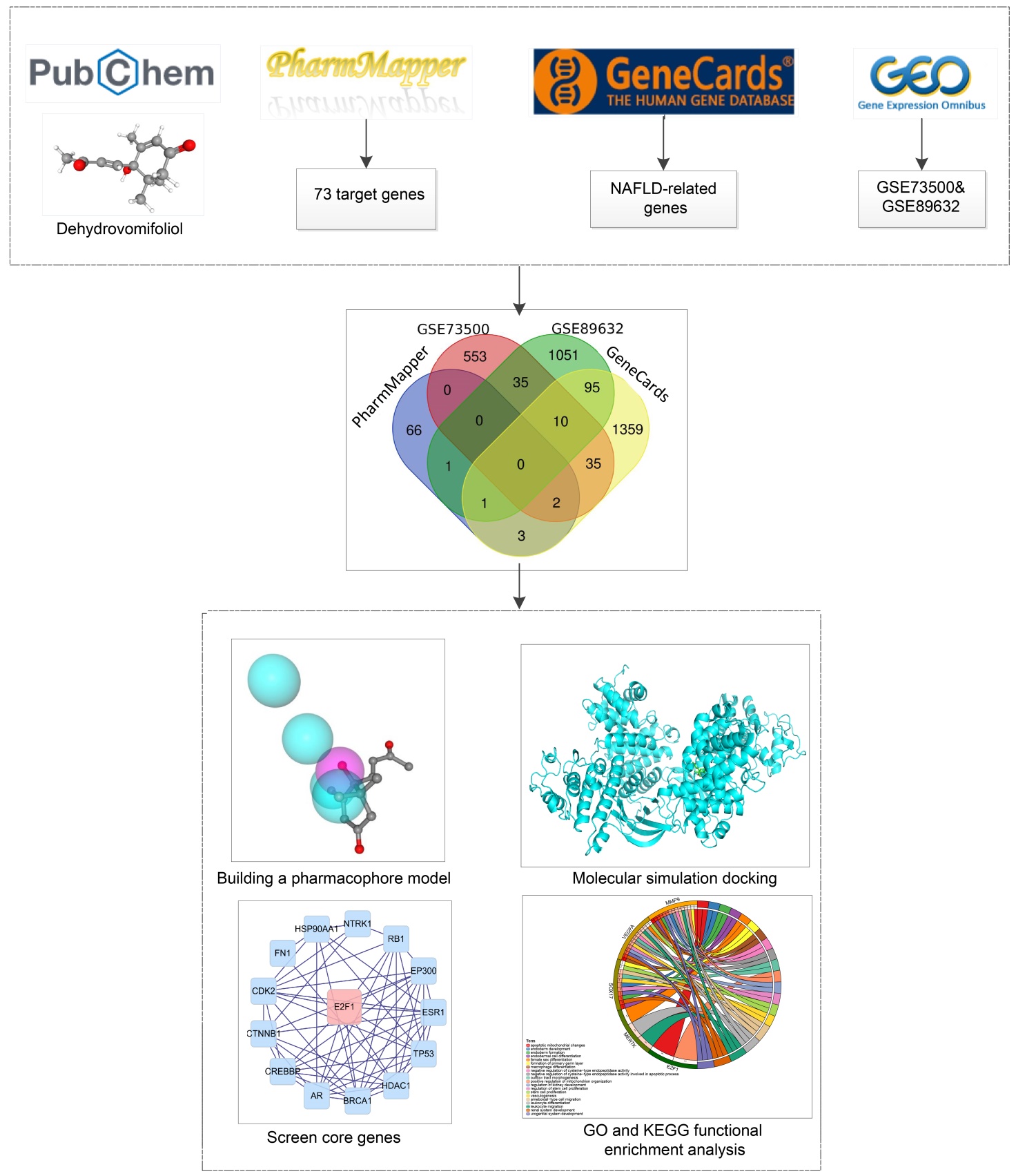


**Figure S1** Protocols of the present study.
